# Change in economic burden of diarrhoea in children under-five in Bangladesh: 2007 vs. 2018

**DOI:** 10.7189/jogh.13.04089

**Published:** 2023-08-25

**Authors:** May Phyu Sin, Md Zahid Hasan, Birger C Forsberg

**Affiliations:** 1 Department of Global Public Health, Karolinska Institutet, Stockholm, Sweden; 2 Health Systems and Population Studies Division, ICDDR,B, Mohakhali, Dhaka, Bangladesh; 3 Nuffield Centre for International Health and Development, Leeds Institute of Health Sciences, University of Leeds, Leeds, UK

## Abstract

**Background:**

In Bangladesh, diarrhoea in children under-five is a major public health problem with cost implications. Although under-five diarrhoea mortality and morbidity have declined from 2007 to 2018, change in the economic burden is unknown. This study determined the change in the societal economic burden of under-five diarrhoea in Bangladesh comparing 2007 to 2018.

**Methods:**

A prevalence-based, retrospective cost analysis was conducted from a societal perspective, including costs to households, providers, and economic loss from premature deaths. Data were obtained from the previous cost of illness studies, government reports, and international databases. Direct costs for treatment were estimated by the bottom-up costing approach. Indirect costs on the loss of productivity of caretakers and loss from premature deaths were calculated by the human capital method. Total costs were presented in both local currency (Bangladeshi Taka (BDT)) and US dollars (US$)) in 2018 price. Sensitivity analyses were conducted to assess the robustness of the input parameters.

**Results:**

A 36.4% reduction was found on the economic burden of under-five diarrhoea when comparing 2007 and 2018; US$1 209 million (95% CI = 1066 million-1299 million) for 2007 and US$769 million (95% CI = 484 million-873 million) for 2018. Economic loss from premature deaths imposed the highest costs (2007 = 66%, 2018 = 66% of all) followed by indirect costs on the loss of productivity of caretakers (2007 = 21%, 2018 = 26%) and direct medical costs (2007 = 13%, 2018 = 8%).

**Conclusions:**

Societal costs from diarrhoeal diseases were reduced from 2007 to 2018 in Bangladesh. However, the economic burden was equivalent to 0.29% of country’s gross domestic product in 2018 and remains a challenge. The major contributor to the costs was premature mortality from diarrhoeal diseases. Premature deaths are still prevalent though they to a large extent are avoidable. To further limit the economic burden, under-five diarrhoea mortality and morbidity reduction should be accelerated.

Diarrhoea, defined as three or more loose stools per day [1], in children under five years of age (under-five diarrhoea) is one of the leading causes of the disease burden. It remains one of the leading causes of premature deaths in children under-five in Bangladesh, with a mortality rate of 12.69 deaths per 100 000 children in 2019 [2]. The estimated number of under-five diarrhoea episodes in Bangladesh was 20 million in 2016, having 1.40 incidence per 1000 person years [3].

Diarrhoea in children under-five has economic implications on households and society in Bangladesh [4-12]. The estimated household costs of diarrhoea per episode in children under-five ranged from US dollars (US$) 1.81 [8] to US$35.40 [10] in 2007. In 2015-2016, the average cost to caregivers ranged from US$6.36 [12] to US$14.94 [11] for outpatient (non-severe) case and US$27.39 [12] to US$56.14 [11] for the hospitalised (severe) case. The costs incurred to the whole society comprising both provider and household costs were estimated at US$8 million in 2007 [7] and US$79 million in 2018 [4].

Previous studies conducted from a societal perspective (**Table 1**) focused on the treatment cost burden by providers, the out-of-pocket expenditure and costs of lost productivity of households, but cost burden of untreated cases or premature deaths were under-represented [4-7]. Furthermore, a direct comparison of the economic burden between the studies is difficult due to differences in the study population, costing approach, type of cases, and cost components included for analysis. None of the studies have looked at trends in the costs of diarrhoeal diseases. Thus, changes in the economic burden of the disease over time remain unknown.

**Table 1:** Description of cost of illness studies conducted from a societal perspective between 1997 and February 2022 in Bangladesh

Description	World Bank’s Water and Sanitation Program [7]	Ahmed S et al. [5]	Rafferty ER et al. [6]	Hasan MZ et al. [4]
Year of costing	2007	2014	2016	2018
Cost estimates	US$8 million	US$10 million	US$9.6 million	US$79 million
Target population	Under-five, diarrhoea attributable to poor sanitation	Under-five, rotavirus diarrhoea	Infants, cryptosporidiosis diarrhoea	Under-five
Costing approach	Prevalence-based	Prevalence-based	Incidence-based	Incidence-based
Type of cases included	Non-hospitalised	Hospitalised	Hospitalised and non-hospitalised	Hospitalised and non-hospitalised
Economic loss from premature deaths	Included	Not included	Included	Not included

Understanding how the economic burden of diarrhoea in children under-five evolves over time in Bangladesh will support policymakers in making effective resource allocation decisions. Thus, this study aimed to fill some of that knowledge gap by estimating the change in the economic burden of diarrhoea in children under-five in Bangladesh from 2007 to 2018.

## METHODS

This is a prevalence-based, retrospective cost analysis study of diarrhoeal diseases in children below five years of age. Costing was conducted from a societal perspective. As the study only used secondary data, no ethical approval was needed.

### Study setting

Bangladesh has a pluralistic health care system where health care is provided by public, private for profits, and private non-profit organisations in the formal sector [13] and village doctors or traditional healers in the informal sector [14,15]. Pharmacies or drug stores are often the first access points for care for both rural and urban populations [13,14].

### Study population

The study population was children under-five years of age who had an episode of acute diarrhoea lasting less than 14 days in Bangladesh in 2007 and 2018, regardless of geographical location, socioeconomic conditions, or disease severity. The years 2007 and 2018 were selected because most data were obtainable for these years.

### Data sources

Under-five diarrhoea prevalence data were obtained from the Bangladesh Demographic and Health Survey (BDHS) reports [16,17]. World Health Organization (WHO) and the Maternal Child Epidemiology Estimation group’s diarrhoea-specific mortality estimates were obtained from the Global Health Observatory database [18]. Cost data for 2007 were mainly identified from the data set of the Global Enteric Multicentre Study (GEMS)-1 Healthcare Services Utilization and Attitudes Survey (HUAS) / HUAS Lite Survey [19]. Cost data for 2018 were mainly identified from the Decade of Vaccine Economics cost of illness database for Bangladesh [20]. Other cost data sources comprised the previous cost of illness studies in Bangladesh [4-6,8-12,21] and WHO-CHOICE unit cost estimates for service delivery [22]. Referenced values, including data sources and the assumptions made, are presented in Table S1.1 to S1.3 in the **Online Supplementary Document**.

### Cost analysis framework

Costing was conducted from a societal perspective, including costs borne by all relevant members in the society as it is considered the most comprehensive method in estimating the economic burden of the disease [23]. This study determined societal costs by referring to the cost categories (medical, non-medical, morbidity, and mortality costs) outlined in the article by Jo [23]. These costs encompassed medical and non-medical expenses associated with seeking and receiving care for diarrheal diseases, caretakers' productivity loss due to caring for sick children (morbidity costs), and economic losses resulting from premature mortality in children under-five due to diarrheal diseases.

**Figure 1:**
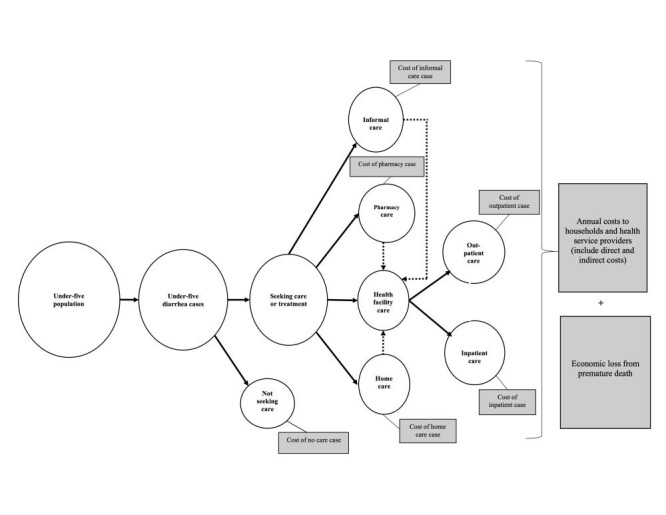
Conceptual framework guiding cost analysis. Based on the health care-seeking patterns of diarrhoea in children under-five in Bangladesh, the study population was stratified into six care prototypes (i) informal care, (ii) pharmacy care, (iii) homecare, (iv) health facility care including outpatient and inpatient care, and (v) no care. As a child may seek care from more than one type of provider, the cases were not mutually exclusive. The costs to households and health service providers were estimated for each care prototype. The costs of each care prototype were summed up to obtain the total annual costs. The economic burden was calculated by summating the total annual costs to households and health service providers and economic loss from premature deaths. The same framework applied to 2007 and 2018 cost calculation.

Based on the local health care seeking patterns for diarrhoea in children under-five [14-16], the study population was stratified into six care prototypes (**Figure 1**): informal care, pharmacy, homecare, health facility including outpatient and inpatient, and no care cases. For this study, no care cases refer to cases for whom no care was sought for the illness; informal care cases denote cases seeking care from the informal sector (village doctors or traditional healers); pharmacy cases are cases seeking care from pharmacies / drug stores [13]; homecare cases are cases taking care at home (self-prescribing) [10]; and health facility cases are cases seeking care at health facilities or qualified health care providers where those treated at health facility outpatient departments or clinics or by community health workers are regarded as outpatient cases and those that were kept in the hospital overnight were counted as inpatient cases. Follow-up cases after hospital discharge were not included in the analyses.

For this study, no care and homecare cases are described as having diarrhoea with no dehydration (mild case). Pharmacy, informal care, and outpatient cases are regarded as diarrhoea with some dehydration (moderate case). Inpatient cases are considered as diarrhoea with severe dehydration (severe case).

### Economic burden estimation

Annual costs to households and health service providers were estimated by 1) calculating cost per diarrhoea episode individually for six care prototypes, 2) calculating the annual costs of each care prototype, and 3) summation of the annual costs of six care prototypes to obtain the total annual costs.

Input parameters for calculating the total annual costs are presented in Table S2 in the **Online Supplementary Document**. Annual costs to households and health service providers were presented by average (base case), minimum, maximum, and 95% confidence interval (CI) values.

The economic loss from premature deaths was calculated using the human capital approach. The economic loss was calculated as future loss of earnings [23], adapting the formula used in Rafferty ER, et al. [6]. Economic loss from premature deaths was calculated as follows:



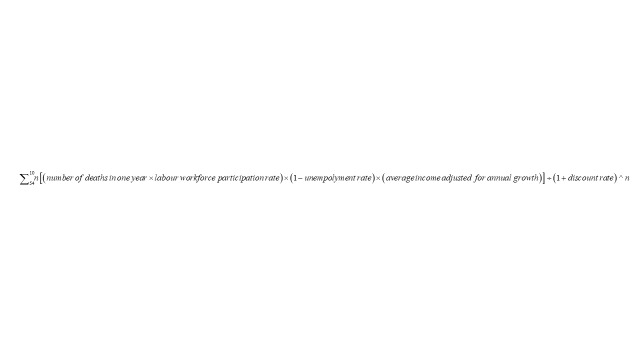



n_10 to 54_ = value between year of earning starts (next ten years from 2007 / 2018, at the age of 14) and year of earning ends (next 55 years from 2007 / 2018, at the age of 59).

Premature deaths were assumed to occur by the age of four, and the loss of earnings was considered to commence ten years later when the child reached a minimum employment age of 14 [24]. Earnings loss due to premature deaths was estimated using the annual average income per person in Bangladesh. To account for wage rate growth, an annual average wage growth of 6% was calculated for both 2007 and 2018, based on the average wage growth from 2010 to 2018 [25].

Future loss of income for a total of 45 years, i.e., legal minimum working age from 14 years [24] to government employee’s retirement age of 59 years [26], was discounted by applying a 3% discount rate annually, a commonly applied discount rate in previous costing studies in Bangladesh [6,7]. The annual average wage growth, labour workforce participation and unemployment rates were also adjusted for both years. Input parameters used are presented in Table S3 in the **Online Supplementary Document**.

### Data analysis

Cost analysis was conducted in Microsoft Excel (Version 16.55). Deterministic and probabilistic sensitivity analyses were conducted using Visual Basic for Applications coding in Excel. Descriptive statistics were used for the subgroup analysis based on disease severity, and the results were presented in terms of cost and percent out of total costs.

### Estimating diarrhoea cases

The period prevalence reported by the BDHS (9.8% in 2007 and 4.7% in 2018) [16,17] was multiplied with an annualisation factor of 52 / 2.5 (52 denotes 52 weeks / one year and 2.5 denotes the number of survey weeks) to obtain the one-year rate [7]. The one-year rate was then applied to the United Nations Population Division’s estimates of under-five populations in Bangladesh [27] for 2007 and 2018 to obtain the total number of under-five diarrhoea cases for each year. The number of cases in each care prototype for 2007 and 2018 was estimated by applying the distribution of children seeking care from each group reported in the BDHS surveys [16,17] to the total estimated number of under-five diarrhoea cases in 2007 and 2018.

The inpatient-to-outpatient ratio among cases seeking care at health facilities was reported as 1:26 in the previous study [6]. For both years, the ratio was assumed to be consistent at 1:26. A sensitivity analysis was conducted to assess the impact of variation from this assumption by testing different percentages of outpatient and inpatient cases.

### Valuation of resources and costs

**Figure 2:**
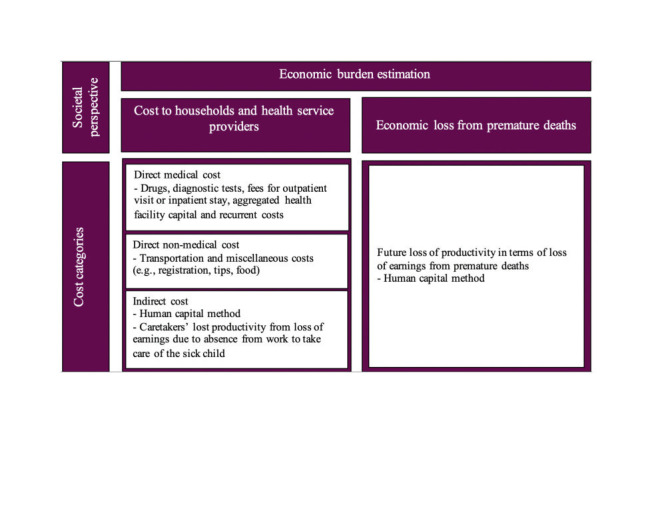
Valuation of resources and costs. The diagram shows different cost categories (direct medical, non-medical, indirect, and economic loss from premature deaths) considered in this study.

Costs were disaggregated into three categories [23] direct medical costs, direct non-medical costs, and indirect costs, as shown in **Figure 2**. The bottom-up costing method [23] was employed to estimate the direct costs if the estimates were not readily available from existing studies.

Indirect costs were calculated using the human capital method [23] by multiplying the total time loss (in days) for caring one episode of diarrhoea in children under-five with the legal minimum wage rate in Bangladesh [24]. Due to limited data on caretakers’ time loss for non-hospitalised cases, 50% of the average duration of mild and moderate diarrhoea in children under-five was used as a proxy for caretakers’ time loss.

Unpaid caregiving times, whether by earning or non-earning caregivers, are considered opportunity costs [28], representing the benefits foregone when they have to care for sick children. To limit the overestimation of costs, the minimum wage rate was used instead of occupation-specific or average wage rates due to the potential variability in caretakers' education status and occupation [16,17]. To ensure data comparability across different years, costs were extracted from sources that utilised the bottom-up (ingredients-based) costing approach. The bottom-up costing approach involves identifying and valuing the resources required for each specific cost item related to the disease of interest using survey questionnaires, interviews, or observations [29]. Specifications were established for each cost item to standardise the data extraction process for the cost of care in both years. The specifications are presented in Table S4 in the **Online Supplementary Document**. Mean values and corresponding 95% confidence intervals (CI) were extracted whenever possible.

The cost estimates were presented in the local currency unit, Bangladeshi Taka (BDT) and US$ in 2018 price. The currency exchange rate referenced from previous studies was BDT69.00 per US$1 in 2007 [10] and BDT83.50 per US$1 in 2018 [4]. The currency conversion rates, and consumer price index (CPI) used in this study are presented in Table S1.4 and S1.5 in the **Online Supplementary Document**.

### Sensitivity analysis

Deterministic sensitivity analysis was performed for both years to identify the key parameters impacting the economic burden estimates and to assess the influence of changes in assumptions and data used for the base case calculation. For cost parameters, the effects of minimum and maximum cost per diarrhoea episode estimate for the six care prototypes were tested. For the labour workforce participation and unemployment rate, minimum and maximum values observed over three decades (1991-2020) were tested. For annual wage growth rate, minimum and maximum values observed between 2010 and 2018 were examined. Effects of different discount rates (0% and 5%) on future income loss were explored. The remaining non-cost parameters were tested by varying them by ±20% from the base case values.

Probabilistic sensitivity analysis (1000 Monte Carlo simulations) [23] was performed to demonstrate the range of uncertainties in the output parameters (annual cost estimates of six care prototypes, total annual costs, and economic loss from premature deaths) in relation to uncertainties in the input parameters. The analysis utilised gamma distribution for cost data and beta distribution for non-cost data [23]. The 2.5th and 97.5th percentile values of the simulated output parameters were reported as 95% CI of that parameter.

Input parameter values used for sensitivity analyses are presented in Table S5.1 to S5.4 in the **Online Supplementary Document**.

## RESULTS

### Cost per diarrhoea episode for six care prototypes

Among the diarrhoea cases who sought care, the cost per diarrhoea episode in children under-five was highest in inpatient case and outpatient cases and was lowest in homecare cases in both 2007 and 2018 (**Table 2**). No care case had a cost per diarrhoea episode of BDT340.62 in 2007 and BDT716.67 in 2018.

**Table 2:** Cost per diarrhoea episode in children under-five by six care prototypes in Bangladesh in 2007 and 2018*

Types of cases	2007 cost per diarrhoea episode†			2018 cost per diarrhoea episode		
	**Base**	**Min**	**Max**	**Base**	**Min**	**Max**
Informal care	643.24	514.60	771.89	1133.89	909.79	1358.00
Pharmacy	605.09	484.08	726.11	1243.80	995.04	1492.56
Homecare	534.96	427.97	641.96	821.74	589.13	1054.34
Outpatient	1689.75	1372.98	2006.51	1736.34	1354.33	2118.34
Inpatient	3944.33	3155.46	4733.19	4483.66	3728.04	5239.27
No care	340.62	272.50	408.75	716.67	573.33	860.00

Min – minimum, Max – maximum

*All currency in local currency unit – Bangladeshi Taka (BDT) in 2018 price.

†2007 costs in 2018 value, inflation adjusted by using the general consumer price index 170.16 in 2018 and 80.55 in 2007. Referenced consumer price indices are presented in the **Online Supplementary Document**.

**Figure 3:**
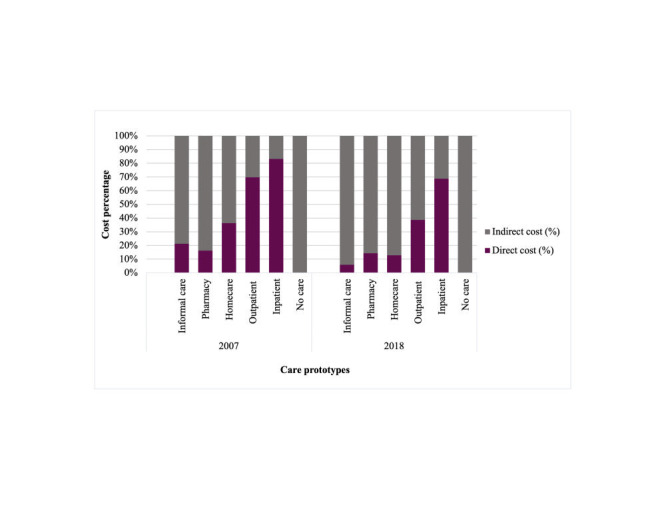
Percent of direct and indirect costs out of total costs per diarrhoea episode for six care prototypes in 2007 and 2018.

The percent distribution of direct and indirect costs out of total costs per diarrhoea episode was found to vary across different care prototypes. In 2007, indirect costs were higher than direct costs in all cases except outpatient and inpatient cases, where the direct costs were 70% and 83% of total costs. Similarly, in 2018, indirect costs were more than direct costs in all cases except in inpatient cases, where the direct costs accounted for 69% of the total costs (**Figure 3**).

### Annual costs to households and health service providers

The annual number of under-five diarrhoea cases in Bangladesh was estimated as 32 433 961 for 2007 and 14 191 441 for 2018, signifying a decline in the total number of cases by 56.2%. The incidence per case (the number of under-five diarrhoea cases divided by the number of under-five population) was estimated to be 2 per case in 2007 and 1 per case in 2018.

**Figure 4:**
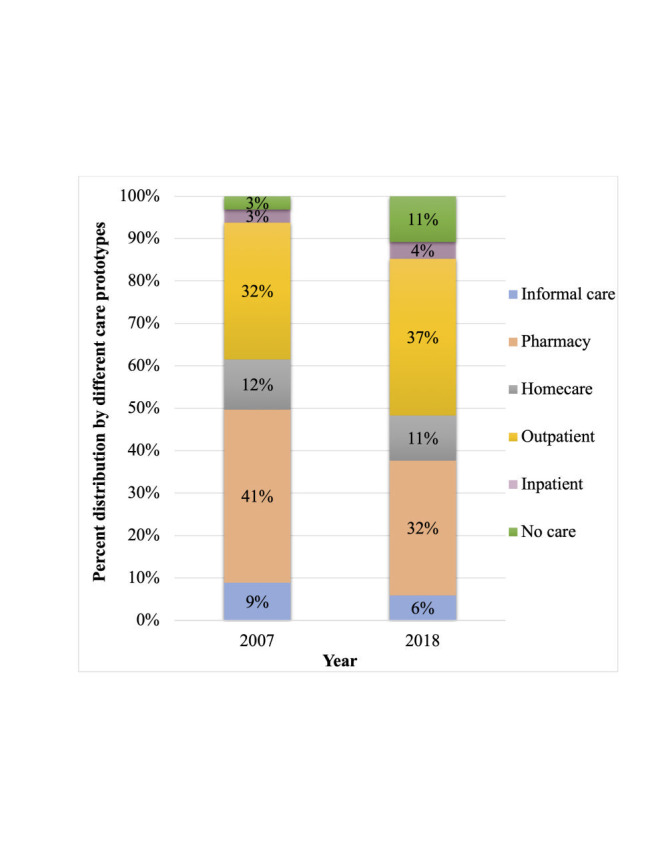
Percent distribution of annual costs to households and health service providers from diarrheal diseases in children under-five in Bangladesh by six care prototypes in 2007 and 2018.

The inflation-adjusted average annual costs to households and health service providers from diarrheal diseases in children under-five were estimated to be BDT33 935 million (US$406 million) in 2007 and BDT21 648 million (US$259 million) in 2018, showing 36.2% decline from 2007 to 2018 (**Table 3**). Similarly, the breakdown of annual household and health service provider costs of each care prototype also decreased from 2007 to 2018 except for no care case where the costs more than doubled in 2018 compared to 2007 (Table S5.1 in the **Online Supplementary Document**). The pharmacy and outpatient cases contributed the most to the cost burden in both 2007 and 2008 (**Figure 4**). Inpatient cases contributed the lowest annual cost burden in both years (3% and 4% respectively).

**Table 3:** Change in annual costs to households and health service providers and economic loss from premature deaths from diarrheal diseases in children under-five in Bangladesh from 2007 to 2018

Description	Annual costs to households and providers		Economic loss from premature deaths	
	In million BDT*	In million US$*	In million BDT*	In million US$*
	**Base (95% CI†)**	**Base (95% CI†)**	**Base (95% CI†)**	**Base (95% CI†)**
2007 costs	16 064 (10 768-19 178)	233 (156-278)	31 726 (29 738-33 829)	460 (431-490)
2007 costs (inflated to 2018 values)‡	33 935 (22 746-40 511)	406 (272-485)	67 018 (62 818-71 460)	803 (752-856)
2018 costs	21 648 (13 661-30 049)	259 (164-360)	42 560 (40 040-45 335)	510 (480-543)
Difference between 2007 and 2018	-12 287	-147	-24 458	-293
Percent change from 2007 to 2018	-36.2%	-36.2%	-36.5%	-36.5%

US$ – US dollar, BDT – Bangladeshi Taka, CI – confidence interval

*The currency exchange rate is BDT69.00 per US$1 in 2007 [10] and BDT83.50 per US$1 in 2018 [4].

†95% CI generated by probabilistic sensitivity analyses (1000 Monte Carlo simulations).

‡Inflation adjusted by using the general consumer price index 170.16 in 2018 and 80.55 in 2007. Referenced consumer price indices are presented in the **Online Supplementary Document**.

Regarding the cost burden based on disease severity levels, moderate cases (cases with some dehydration) imposed the largest cost burden on households and health service providers in both years (**Table 4**). The cost burden from severe cases (cases with severe dehydration) as defined in this study only amounted to 3% to 4% of total costs.

**Table 4:** Annual costs to households and health service providers from diarrheal diseases in children under-five in Bangladesh in 2007 and 2018 based on disease severity

Types of case	2007 annual costs* (million BDT)	Percent out of total annual costs	2018 annual costs (million BDT)	Percent out of total annual costs
Mild cases	2402	15%	4637	21%
Moderate cases	13 158	82%	16 149	75%
Severe cases	503	3%	860	4%
All cases	16 064	100%	21 648	100%

BDT – Bangladeshi Taka

*2007 annual costs are in 2007 price (inflation unadjusted values).

### Economic loss from premature deaths

The estimated number of deaths from diarrhoea in children under-five in Bangladesh was 18 438 deaths in 2007 and 6751 in 2018. The inflation-adjusted economic loss from premature deaths due to diarrheal diseases in children under-five in Bangladesh in 2007 was estimated as BDT67 018 million (US$803 million). The economic loss from premature deaths decreased by 36.5% in 2018, amounting to BDT42 560 million (US$510 million) (**Table 3**).

### Economic burden estimates

The inflation-adjusted total economic burden of diarrhoea in children under-five in Bangladesh was found to be BDT100 954 million (US$1 209 million) in 2007 and BDT64 208 million (US$769 million) in 2018 (**Table 5**). The overall economic burden of diarrhoea in children under-five in Bangladesh decreased by 36.4% from 2007 to 2018.

**Table 5:** Change in total economic burden of diarrhoea in children under-five in Bangladesh from 2007 to 2018

Description	Total economic burden (million BDT*)	Total economic burden (million US$*)
	**Base (95% CI†)**	**Base (95% CI†)**
2007 costs	47 791 (42 134-51 336)	693 (611-744)
2007 costs (inflated to 2018 values)‡	100 954 (89 003-108 442)	1209 (1066-1299)
2018 costs	64 208 (40 455-72 923)	769 (484-873)
Difference between 2007 and 2018	-36745	-440
Percent change from 2007 to 2018	-36.4%	-36.4%

BDT – Bangladeshi Taka, US$ – US dollar

*The currency exchange rate is BDT69.00 per US$1 in 2007 [10] and BDT83.50 per US$1 in 2018 [4].

†95% CI generated by probabilistic sensitivity analyses (1000 Monte Carlo simulations).

‡Inflation adjusted by using general consumer price index 170.16 in 2018 and 80.55 in 2007. Referenced consumer price indices are presented in the **Online Supplementary Document**.

For both 2007 and 2018, the major cost attributing to the total economic burden of diarrhoea in children under-five was the economic loss from premature deaths, accounting for 66% of the total economic burden in both years. Indirect costs (lost productivity costs) were responsible for 21% and 26% of the economic burden of the disease in 2007 and 2018 respectively. Direct medical and non-medical costs only contributed to 13% of the economic burden of the disease in 2007 and 8% in 2018. The detailed costs are presented in Table S6.2 in the **Online Supplementary Document****.**

### Deterministic sensitivity analysis results

**Figure 5:**
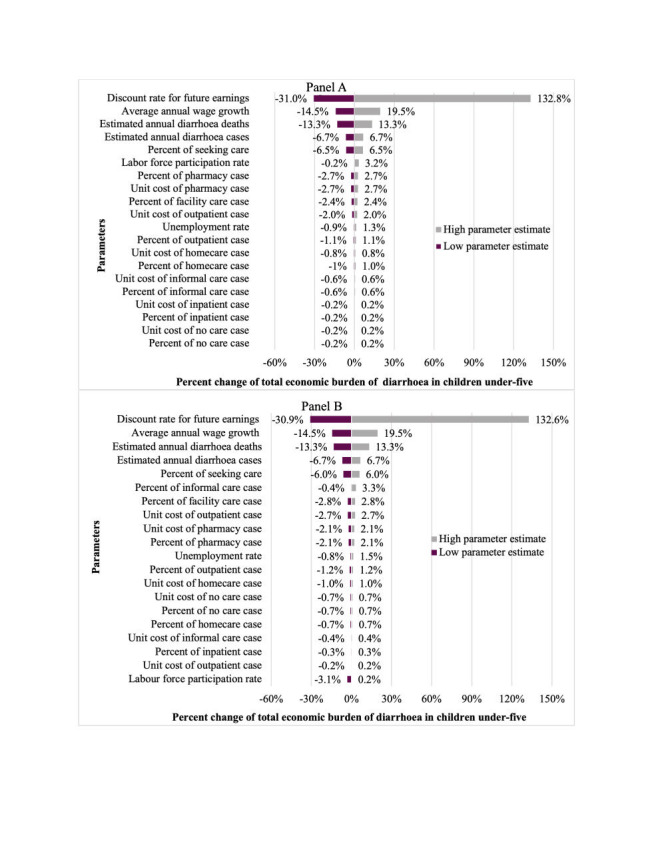
Sensitivity analysis of input parameters applied in calculating the economic burden of diarrhoea in children under-five in Bangladesh. The tornado diagram displays the percent increase or decrease in total economic burden estimate from variations of inputs to economic burden estimation. **Panel A.** Year 2007. **Panel B.** Year 2018.

Variation in following five parameters were found to have the largest impact on the economic burden of diarrhoea in children under-five in both 2007 and 2018 **(****Figure 5****)**: 1) discount rate (zero and five percent) used for estimating present values of future earnings, 2) average annual wage growth rate (five and seven percent), 3) estimated number of diarrheal deaths in one year (±20% from the base case), 4) estimated number of diarrhoea cases in one year (±20% from the base case) and 5) percent of cases seeking care (±20% from the base case).

## DISCUSSION

The findings suggest that the economic burden of diarrhoea in children under-five in Bangladesh was reduced by 36.4% from 2007 to 2018. The total annual costs to households and health service providers were decreased by 36.2%, whereas the economic loss to society from premature deaths was reduced by 36.5% in 2018 compared to 2007. The economic burden was equivalent to 0.87% and 0.29% of the country’s gross domestic product (GDP) in 2007 and 2018 respectively.

The decline in the economic burden primarily occurred due to decreased morbidity and mortality burden of diarrhoea in under-five population in Bangladesh, which may be attributable to improved water, sanitation, and hygiene conditions, an increase in exclusive breastfeeding rate and improved coverage of basic effective treatment (ORT and zinc) for treating under-five diarrhoea in the country [16,17,30]. From 2007 to 2018, the percentage of people using improved sanitation facilities increased from 25% to 39% [31]; access to clean water increased from 55% to 58% [32]; exclusive breastfeeding rate increased from 42.9% to 65% [30], and the combined ORT and zinc treatment coverage doubled from 20% to 44% [16].

There was only 36.4% reduction in the economic burden of disease from 2007 to 2018 although there were 56% reduction in under-five diarrhoea cases and 63% reduction in premature deaths. This disproportionate decline is due to higher wage rates in 2018, resulting in higher indirect cost estimates, increased future productivity value of each child, and higher economic loss from premature deaths in 2018.

Another factor is an increase in the proportion of health facilities cases (23% in 2007 vs. 44% in 2018) [7,16]. As the costs for seeking care at health facilities are higher than those for seeking care from other types of providers [6], the reduction in total cost burden from fewer cases may be offset by increased demand for more costly health facility care in 2018. This change in health care seeking behaviour may be a reflection of higher relative income in households.

The economic loss from premature deaths was the main contributor to the economic burden of diarrhoea in children under-five which is consistent with previous studies in Bangladesh [6,7]. However, the 2018 estimated costs to households and providers from this study is higher than the study by Hasan et al. [4]; US$259 million vs. US$79 million. As 23% of under-five children with diarrhoea were reported not seeking care from any providers in 2018 [16], including untreated cases in this study yields higher cost estimates.

Similarly, the 2007 estimates of treatment and costs of lost productivity of caretakers is higher than found in the previous study [7]; BDT33 935 million vs. BDT16 586 million. This is because the previous study estimated the economic impacts of diarrhoea attributable to inadequate sanitation only [7], while this study estimated the total economic burden of diarrhoea, reflecting the impact from all risk factors.

It is a strength of the study that the cost analysis was based on local data sources and a nationally representative BDHS, resulting in increased generalisability of the study findings. Additionally, changes in the country’s wage growth, labour workforce participation, and unemployment rate over time were reflected and adjusted in estimating the future loss of earnings of children under-five from premature deaths from diarrheal diseases, thereby limiting the overestimation of economic loss from premature deaths. Lastly, the cost analysis was based on local health care seeking patterns specific to diarrhoea in children under-five, including diarrhoea cases at health facilities and in the community. Thus, findings represent a comprehensive societal economic burden of under-five diarrhoea in Bangladesh.

While this study has provided valuable insights into the change in the economic burden of childhood diarrhoeal diseases in Bangladesh, certain limitations need to be acknowledged. The number of diarrhoea cases and deaths were found to be the main cost drivers of economic burden estimates in both years in deterministic sensitivity analyses. Thus, misreporting of diarrhoea cases and misclassifying the causes of death can result in over or underestimation of cost estimates. However, choosing reliable data sources helps to increase the study's internal validity. Data collection for BDHS reports occurred during peak seasons, i.e., summer and winter times [32]. Annualising diarrhoea prevalence without adjusting for seasonal variability might overestimate the diarrhoea cases in both years.

The costs were prone to the confounding effects of other disease comorbidities. Using the data from the bottom-up costing approach enables a focused assessment of health care costs specific to the disease under study [33], reducing the influence of comorbidities on the collected health expenses. Nonetheless, potential confounding effects cannot be completely eliminated. Additionally, external factors like changes in hospital policies and the country's economy, which were not considered in this study, can impact the economic burden of the disease in 2007 and 2018.

The human capital method used in this study can overestimate the value of foregone productivity of caretakers compared to the alternative friction cost method [23]. However, the risk of overestimation was limited by using the minimum wage rate rather than the average wage rate.

The direct cost estimates are subject to measurement bias due to variations in study designs and data collection processes of primary costing studies across different years. However, its impact on the study results was deemed minimal, considering that direct costs constituted the smallest proportion among the cost categories of the economic burden of diarrhoea (13% in 2007 and 8% in 2018). Additionally, deterministic sensitivity analyses indicated that variations in cost parameters had a lesser impact on the economic burden estimates compared to non-cost parameters in both years.

Using standardised cost item definitions and data from bottom-up costing studies for both years enhances the comparability of direct costs between 2007 and 2018. Nonetheless, standardised data collection processes and costing are essential to provide more comparable evidence of health care costs across different time frames in the future.

This study has policy implications at the national level. Sustained funding and collaborative efforts on implementing preventive and treatment interventions are much needed to further reduce the morbidity, mortality, and economic burden of childhood diarrheal diseases. As a 20% reduction in premature deaths in 2018 can result in savings of BDT8 512 million as estimated by this study, policymakers should consider allocating the financial resources to the most cost-effective interventions that can bring down under-five diarrhoea mortality. Increasing the universal health coverage (UHC) can help to reduce the economic burden of diarrhoea in children as the essential health service package covered in the UHC contains several high-impact interventions such as strengthening diarrhoea case management (ORS and zinc usage) and promoting exclusive breastfeeding that can reduce diarrhoea morbidity and mortality in under-five population [34]. Future studies on the economic impacts of childhood diarrheal diseases focused on risk groups, e.g., children living in slums or endemic areas, will be beneficial to identify target areas and populations for interventions.

## CONCLUSIONS

This study can inform the policymakers that the economic burden of diarrhoea in children under-five in Bangladesh was reduced by approximately a third (36.4%) from 2007 to 2018. However, the economic burden of diarrhoea in children under-five remains substantial, amounting to BDT64 208 million (US$769 million), equivalent to 0.29% of country’s GDP in 2018. The economic burden is driven mainly by the economic loss from premature deaths. Thus, the most cost-effective diarrhoea prevention, control and treatment interventions need to be identified and scaled up. UHC should be increased to accelerate the reduction in the mortality and morbidity of diarrhoea, which will ultimately reduce the economic burden of diarrhoea in children under-five and release resources for other pressing needs.

## Additional material


Online Supplementary Document
