# Supplementary material for: Change in economic burden of diarrhoea in children under-five in Bangladesh: 2007 vs. 2018
Source: J Glob Health. 2023 Aug 25;13:04089. doi: 10.7189/jogh.13.04089 (PMC10451101; doi:10.7189/jogh.13.04089)
Supplement: Online Supplementary Document [file jogh-13-04089-s001.pdf]

Change in economic burden of diarrhoea in children under-five in Bangladesh: 2007 versus 2018

Online Supplementary Document

Appendix S1: Price and resources used for estimating costs per diarrhoea episode for six care prototypes

Table S1.1. Non-cost parameters used for estimating costs per diarrhoea episode for six care prototypes for 2007 and 2018

Table S1.2. Cost parameters used for estimating costs per diarrhoea episode for six care prototypes for 2007

Table S1.3. Cost parameters used for estimating cost per diarrhoea episode for six care prototypes for 2018

Table S1.4. Currency exchange rate used in cost calculation

Table S1.5. Consumer Price indices (CPI) used in cost calculation

Appendix S2: Input parameters for estimating annual costs to households and health service providers

Table S2. Input parameters for estimating annual costs to households and health service providers from diarrheal diseases in children under-five in Bangladesh in 2007 and 2018

Appendix S3: Input parameters for estimating economic loss from premature deaths

Table S3. Input parameters for estimating economic loss from premature deaths from diarrhoeal diseases in children under-five in Bangladesh for 2007 and 2018

Appendix S4: Cost item specifications

Table S4. Specifications of each cost item for standardized data extraction for the cost of care in 2007 and 2018

Appendix S5: Input parameters for sensitivity analyses

Table S5.1. Input parameters values for deterministic (one-way sensitivity analysis) for 2007

Table S5.2. Input parameters values for deterministic (one-way sensitivity analysis) for 2018

Table S5.3. Input parameters values for probabilistic sensitivity analysis for 2007

Table S5.4. Input parameters values for probabilistic sensitivity analysis for 2018

Appendix S6: Cost breakdown

Table S6.1. Annual costs to households and health service providers from diarrhoea in children under-five in Bangladesh in 2007 and 2018 by six care prototypes and percent change in annual costs from 2007 to 2018

Table S6.2. Cost breakdown of total economic burden of the diarrhoea in children under-five in Bangladesh in 2007 and 2018

References

Appendix S1: Price and resources used for estimating costs per diarrhoea episode for six care prototypes

Table S1.1. Non-cost parameters used for estimating costs per diarrhoea episode for six care prototypes for 2007 and 2018

Non-cost parameters	2007	Source	2018	Source
Hospital length of stay (days)	4.20	[1]	4.20	[2]
Duration of diarrhoea in mild case (days)	4.30	[3]	4.30	[3]
Duration of diarrhoea in moderate case (days)	6.40	[3]	6.40	[3]
Duration of diarrhoea in servere case (days)	8.40	[3]	8.40	[3]
Legal monthly minimum wage (in BDT)	1800.00	[4]	8000	[5]
Number of working days per month	24.00	[4]	24.00	[4]
Minimum wage per day (Legal monthly minimum wage divided by number of working days per month)	75.00	..	333.33	..

Table S1.2. Cost parameters used for estimating costs per diarrhoea episode for six care prototypes for 2007

Cost parameters	Estimate from literature (Year)	Base (2007 BDT)	Source	Min (2007 BDT)	Max (2007 BDT)	Source
**Informal care case**						
**Direct medical cost**						
Costs on drugs and consultation	BDT 61.71 (2007)	61.71	[6]	49.37	74.05	±20% from base
**Direct non-medical cost**						
Travel cost	BDT 2.8 (2007)	2.80	[6]	2.24	3.36	±20% from base
**Indirect cost**						
Caretakers' time loss (days)		3.20	50% of average duration of diarrhoea in moderate case	2.56	3.84	±20% from base
**Pharmacy case**						
**Direct medical cost**						
Drug and consultation costs	BDT 43 (2007)	43.00	[6]	34.40	51.60	±20% from base
**Direct non-medical cost**						
Travel cost	BDT 3.45 (2007)	3.45	[6]	2.76	4.14	±20% from base
**Indirect cost**						
Caretakers' time loss (days)		3.20	50% of average duration of diarrhoea in moderate case	2.56	3.84	±20% from base
**Homecare case**						
**Direct medical cost**						
Costs for buying remedies/medicine	BDT 92 (2007)	92.00	[7]	73.60	110.40	±20% from base
**Direct non-medical cost**						
Travel cost	BDT 0 (2007)	0.00	[6]	0.00	0.00	..
**Indirect cost**						
Caretakers' time loss (days)		2.15	50% of average duration of diarrhoea in mild case	1.72	2.58	±20% from base
**Outpatient case**						
**Direct medical cost**						
Cos per outpatient visit excluding drugs and diagnostic tests	BDT 74.60, 66.45 (2007)	70.53	Average of min and max costs	66.45	74.60	[8]
Costs on drugs and diagnostic tests	BDT 365 (2007)	365.00	[9]	292.00	438.00	±20% from base
**Direct non-medical cost**						
Travel cost	BDT 100 (2007)	100.00	[6]	80.00	120.00	±20% from base
Miscellaneous costs	BDT 31 (2011)	22.42	[10]	17.93	26.90	±20% from base
**Indirect cost**						
Caretakers' time loss (days)		3.23	50% of average duration of diarrhoea in moderate case	2.58	3.87	±20% from base
**Inpatient case**						
**Direct medical cost**						
Cost per bed day excluding drugs and diagnostic costs (primary and secondary level hospitals*)	BDT 198.6, 207.2 (2007)	202.90	Average cost of primary and secondary hospitals [8]	162.32	243.48	±20% from base
Cost per bed day for hospital stay per diarrhoea episode		852.18	Cost per bed day multiplied by average hospital length of stay	681.74	1022.62	±20% from base
Capital cost on main equipment i.e., microscope		14.36	Author estimates based on previous cost study on district hospital [11]	11.49	17.23	±20% from base
Recurring costs on drugs and lab tests	BDT 76.83 (1997)	124.96	[11]	99.97	149.95	±20% from

Cost parameters	Estimate from literature (Year)	Base (2007 BDT)	Source	Min (2007 BDT)	Max (2007 BDT)	Source
						base
**Direct non-medical cost**						
Travel cost	US$4.00 (2010)	347.59	[12]	278.07	417.10	±20% from base
Miscellaneous costs	US$3.78 (2010)	213.15	[12]	170.52	255.78	±20% from base
**Indirect cost**						
Caretakers' time loss (days)		4.20	Average hospital length of stay	3.36	5.04	±20% from base
		**No care case**				
**Indirect cost**						
Caretakers' time loss (days)		2.15	50% of average duration of diarrhoea in mild case	1.72	2.58	±20% from base

*Primary level hospital denotes upazila health complexes; secondary level hospital denotes district hospitals. All currency in local currency unit - Bangladeshi Taka (BDT). Base, minimum, and maximum costs are in 2007 cost values.

Table S1.3. Cost parameters used for estimating cost per diarrhoea episode for six care prototypes for 2018

Cost parameters	Estimate from literature (Year)	Base (2018 BDT)	Source	Min (2018 BDT)	Max (2018 BDT)	Source
			**Informal care case**			
**Direct medical cost**						
Costs on drugs and consultation	US$0.5 (2011), US$0.65 (2012)	62.03	Average of min [13], max [14] costs	52.30	71.76	Min [13] Max [14]
**Direct non-medical cost**						
Travel cost per diarrhoea episode	BDT 5.2 (2018)	5.20	Assumed same cost as pharmacy case	4.16	6.24	Assumed same cost as pharmacy case
**Indirect cost**						
Caretakers' time loss (days)		3.20	50% of average duration of diarrhea in moderate cases	2.56	3.84	±20% from base
			**Pharmacy case**			
**Direct medical cost**						
Drug and consultation costs	BDT 171.93 (2018)	171.93	[15]	137.54	206.31	±20% from base
**Direct non-medical cost**						
Travel cost per diarrhoea episode	BDT 5.2 (2018)	5.20	[15]	4.16	6.24	±20% from base
**Indirect cost**						
Caretakers' time loss (days)		3.20	50% of average duration of diarrhea in moderate cases	2.56	3.84	±20% from base
			**Homecare case**			
**Direct medical cost**						
Costs for buying remedies/medicine	BDT 92 (2007)	105.07	[7]	15.80	194.34	±20% from base
**Direct non-medical cost**						
Travel cost per diarrhoea episode		0.00	..	0.00	0.00	..
**Indirect cost**						
Caretakers' time loss (days)		2.15	50% of average duration of diarrhoea in mild cases	1.72	2.58	±20% from base
			**Outpatient case**			
**Direct medical cost**						
Cost per outpatient visit including drugs and diagnostic costs	US$6.53 (5.02 to 8.05) (2018)	545.67	[2]	419.17	672.18	[2]
**Direct non-medical cost**						
Travel and miscellaneous costs per diarrhea episode	US$1.49 (0.98 to 1.99) (2018)	124.00	[2]	81.83	166.17	[2]
**Indirect cost**						
Caretakers' time loss (days)		3.20	50% of average duration of diarrhea in moderate cases	2.56	3.84	±20% from base
			**Inpatient care case**			
**Direct medical cost**						
Capital, overhead, labor, drugs and diagnostic cost (primary and secondary level hospitals*)	US$24.48 (Primary level), US$15.58 (Secondary level) (2018)	1672.51	Average cost of primary and secondary level hospitals obtained from Hasan MZ et al [2]	1338.00	2007.01	±20% from base
**Direct non-medical cost**						
Travel and miscellaneous costs per diarrhea episode	US$16.9 (15.21 to 18.59) (2018)	1411.15	[2]	1270.04	1552.27	[2]
**Indirect cost**						
Caretakers' time loss (days)		4.20	Average hospital length of stay	3.36	5.04	±20% from base
			**No care case**			
**Indirect cost**						
Caretakers' time loss (days)		2.15	50% of average duration of diarrhea in mild cases	1.72	2.58	±20% from base

*Primary level hospital denotes upazila health complexes; secondary level hospital denotes district hospitals. All currency in local currency unit - Bangladeshi Taka (BDT). Base, minimum, and maximum costs are in 2018 cost values.

Table S1.4. Currency exchange rate used in cost calculation

Reference for currency conversion	Local currency (BDT) per US$
Currency conversion rate for 2018 from Hasan MZ et al. study [2]	83.50
Currency conversion rate for 2016 from Sultana R et al. study [16]	78.40
Currency conversion rate for 2012 from IMF [17]	81.86
Currency conversion rate for 2011 from IMF [17]	74.15
Currency conversion rate for 2010 from Bhuiyan et al. study [12]	70.00
Currency conversion rate for 2007 from Halder AK et al. study [18]	69.00

Table S1.5. Consumer Price indices (CPI) used in cost calculation

Year	General CPI (Base year 2010)	CPI for medical services (Base year 1995)	CPI for medical services (Base year 2005)
1995	42.53	100.00	
1996	43.55	105.31	
1997	45.86	114.15	
1998	49.71	121.12	
1999	52.74	123.80	
2000	53.91	129.82	
2001	54.99	136.22	
2002	56.82	145.25	
2003	60.04	154.36	
2004	64.60	162.67	
2005	69.15	169.62	100.00
2006	73.83	178.49	105.23
2007	80.55	185.66	109.46
2008	87.73	189.25	111.57
2009	92.48	199.22	117.45
2010	100.00	203.67	144.55
2011	111.40	203.67	152.63
2012	118.32	203.67	159.66
2013	127.23		164.06
2014	136.13		180.77
2015	144.56		199.94
2016	152.53		206.70
2017	161.23		209.28
2018	170.16		215.31

CPIs for medical services were obtained from statistical year books (31st and 40th editions) published by the Bangladesh Bureau of Statistics [19,20]. General CPIs were obtained from International Monetary Fund (IMF) [17].

Appendix S2: Input parameters for estimating annual costs to households and health service providers

Table S2. Input parameters for estimating annual costs to households and health service providers from diarrheal diseases in children under-five in Bangladesh in 2007 and 2018

Parameters	2007		2018	
**Non-cost parameters**	**Base case**	**Source**	**Base case**	**Source**
Under-five children population	15 911 480	[21]	14 516 613	[21]
Under-five diarrhoea prevalence rate	9.8	[22]	4.7	[23]
Annualized under-five diarrhoea prevalent cases	32 433 961	Author estimate*	14 191 441	Author estimate*
Percent sought care from any providers	90	[22]	77	[23]
Percent not sought care from any providers	10	[22]	23	[23]
Percent sought care from informal sector among care seeking cases	16	[9,22]	10	[23]
Percent sought care from pharmacies among care seeking cases	78	[9,22]	51	[23]
Percent received homecare among care seeking cases	26	[24]	26	Assumed same % as 2007
Percent sought care from health facility among care seeking cases	23	[9,22]	44	[23]
Percent of outpatient cases among health facility cases	96	Assumption†	96	Assumption†
Percent of hospitalized cases health facility cases	4	Assumption†	4	Assumption†
**Cost parameters (Cost per diarrhoea episode)**	**Base case (BDT)**	**Source**	**Base case (BDT)**	**Source**
Informal care	304.51	Author estimate	1 133.89	Author estimate
Pharmacy case	286.45	Author estimate	1 243.80	Author estimate
Homecare case	253.25	Author estimate	821.74	Author estimate
Outpatient case	799.92	Author estimate	1 736.34	Author estimate
Inpatient case	1 867.23	Author estimate	4 483.66	Author estimate
No care case	1 61.25	Author estimate	7 16.67	Author estimate

BDT - Bangladeshi Taka.
All currency presented in this table are in local currency unit - BDT. Costs are inflation unadjusted values.
*Estimated by multiplying the two weeks prevalence with annualization factor of 52/2.5
†Percent calculated by applying inpatient and outpatient ratio (1:26) among cases seeking care at health facility referenced in Rafferty ER et al [25].

Appendix S3: Input parameters for estimating economic loss from premature deaths

Table S3. Input parameters for estimating economic loss from premature deaths from diarrhoeal diseases in children under-five in Bangladesh for 2007 and 2018

	2007		2018	
**Parameters**	**Base case**	**Source**	**Base case**	**Source**
Number of under-five diarrhoea deaths	18 438	[26]	6 751	Author estimate*
Minimum age for admission to employment (years)	14	[4]	14	[4]
Retirement age (years)	59	[27]	59	[27]
Working life years (from age 14 to 59)	45	-	45	-
Assumed age at death (years)	4	-	4	-
Labor force participation rate among population aged 15-64 (%)	58.7%	[28]	61.4%	[28]
Unemployment rate (%)	4.1%	[29]	4.3%	[29]
Average monthly income per person (in BDT)	3 779	[30]	13 258	[31]
Average annual income per person (in BDT)	45 348	monthly income*12	15 9096	monthly income* 12
Average annual wage growth (%)	6.0%	[32]	6.0%	[32]
Discount rate for future earnings (%)	3.0%	25,9,33	3.0%	25,9,33

BDT - Bangladeshi Taka

*Total deaths in 2018 were estimated by applying the 2015 under-five diarrhoea-specific mortality rates (2.3 per 1000 live births) estimates by Black, R ect [34] to live birth population in 2018 (2 935 000) reported by United Nations Population Division [21].

Appendix S4: Cost item specifications

Table S4. Specifications of each cost item for standardized data extraction for the cost of care in 2007 and 2018

Medical cost for non-health facility care<br>1. Drug cost: Cost of buying drugs or remedies to treat diarrhoea (self-prescribed or pharmacy-prescribed medications)<br>2. Consultation cost: Fees charged by informal providers
Non-medical cost for non-health facility care<br>1. Travel cost: Costs (round trip) for traveling to care provision sites for one diarrhoea episode
Medical cost for health facility care<br>1. Capital and recurrent costs (excluding drugs and diagnostic costs): Aggregated costs calculated by bottom-up costing approach<br>2. Drugs and diagnostic costs (provider): Drugs and diagnostic costs for treating diarrhoea incurred by the health facility<br>3. Drugs and diagnostic costs (household): Drugs and diagnostic costs for treating diarrhoea paid by the household, also referred to as household out-of-pocket expenditure
Non-medical cost for health facility care<br>1. Travel cost: Total costs (round trip) for traveling to care provision sites for one diarrhoea episode<br>2. Miscellaneous cost: Household costs that are not related to medical care and travel costs e.g., informal payment at health facility (tips) or registration or food cost

Appendix S5: Input parameters for sensitivity analyses

Table S5.1. Input parameters values for deterministic (one-way sensitivity analysis) for 2007

Non-cost parameters	Low Value	High Value	Source
Annualized under-five diarrhea prevalent cases	25 947 169	38 920 753	± 20% from base case
Percent sought care from any providers	72%	100%	± 20% from base case
Percent not sought care from any providers	8%	12%	± 20% from base case
Percent sought care from informal sector	13%	19%	± 20% from base case
Percent sought care from pharmacies	62%	94%	± 20% from base case
Percent received homecare	20%	31%	± 20% from base case
Percent sought care from health facility	18%	28%	± 20% from base case
Percent of outpatient cases	86%	100%	± 20% from base case
Percent of hospitalized cases	3%	5%	± 20% from base case
Number of under-five diarrhoea deaths	14 750	22 126	± 20% from base case
Labor force participation rate	58.5%	61.5%	Lowest and highest value between 1991 & 2020 [28]
Unemployment rate (%)	2.2%	5.4%	Lowest and highest value between 1991 & 2020 [29]
Average annual wage growth (%)	5%	7%	Lowest and highest value between 2010 & 2018 [32]
Discount rate for future earnings (%)	0%	5%	-
**Cost parameters (Cost per diarrhoea episode)**	**Low Value**	**High Value**	**Source**
Informal care case	243.61	365.41	Author estimates
Pharmacy case	229.16	343.74	Author estimates
Homecare case	202.60	303.90	Author estimates
Outpatient case	649.97	949.88	Author estimates
Inpatient case	1 493.79	2 240.68	Author estimates
No care case	129.00	193.50	Author estimates

All currency in local currency unit - Bangladeshi Taka (BDT). Costs are inflation unadjusted values.

Table S5.2. Input parameters values for deterministic (one-way sensitivity analysis) for 2018

Non-cost parameters	Low Value	High Value	Source
Annualized under-five diarrhoea prevalent cases	11 353 153	17 029 729	± 20% from base case
Percent sought care from any providers	62%	92%	± 20% from base case
Percent not sought care from any providers	18%	28%	± 20% from base case
Percent sought care from informal sector	8%	12%	± 20% from base case
Percent sought care from pharmacies	40%	61%	± 20% from base case
Percent received homecare	20%	31%	± 20% from base case
Percent sought care from health facility	35%	53%	± 20% from base case
Percent of outpatient cases	86%	100%	± 10% from base case
Percent of hospitalized cases	3%	5%	± 20% from base case
Number of under-five diarrhoea deaths	5 400	8 101	± 20% from base case
Labor force participation rate	58.5%	61.5%	Lowest and highest value between 1991 & 2020 [28]
Unemployment rate (%)	2.2%	5.4%	Lowest and highest value between 1991 & 2020 [29]
Average annual wage growth (%)	5%	7%	Lowest and highest value between 2010 & 2018 [32]
Discount rate for future earnings (%)	0%	5%	[33]
**Cost parameters (Cost per diarrhoea episode)**	**Low Value**	**High Value**	**Source**
Informal care case	909.79	1358.00	Author estimates
Pharmacy case	995.04	1492.56	Author estimates
Homecare case	589.13	1054.34	Author estimates
Outpatient case	1 354.33	2 118.34	Author estimates
Inpatient case	3 728.04	5 239.27	Author estimates
No care case	573.33	860.00	Author estimates

All currency in local currency unit - Bangladeshi Taka (BDT). Costs are inflation unadjusted values.

Table S5.3. Input parameters values for probabilistic sensitivity analysis for 2007

**Non-cost parameters**	**PSA Value**	**Distribution Function**	**Alpha**	**Beta**	**Mean**	**Variance**
Percent sought care from any providers	0.91	Beta	1.50	0.16	0.90	0.03
Percent not sought care from any providers	0.09	Beta	22.50	211.91	0.10	0.00
Percent sought care from informal sector	0.15	Beta	20.84	109.41	0.16	0.00
Percent sought care from pharmacies	0.63	Beta	4.72	1.33	0.78	0.02
Percent received homecare	0.23	Beta	18.34	53.31	0.26	0.00
Percent sought care from health facility	0.22	Beta	407.10	1 362.90	0.23	0.00
Percent of outpatient cases	0.95	Beta	3.04	0.13	0.96	0.01
Percent of hospitalized cases	0.05	Beta	23.96	575.04	0.04	0.00
Labor force participation rate	0.60	Beta	493.95	347.82	0.59	0.00
Unemployment rate	0.03	Beta	6.20	144.21	0.04	0.00
**Cost parameters (Cost per diarrhoea episode)**	**PSA Value**	**Distribution Function**	**Alpha**	**Beta**	**Mean**	**Variance**
Informal care case	257.51	Gamma	12.50	24.36	304.51	7 418.11
Pharmacy case	269.74	Gamma	12.50	22.92	286.45	6 564.29
Homecare case	275.72	Gamma	12.50	20.26	253.25	5 130.85
Outpatient case	1 090.26	Gamma	14.23	56.22	799.92	44 972.62
Inpatient case	2 353.12	Gamma	12.50	149.37	1 867.23	278 924.81
No care case	170.42	Gamma	12.50	12.90	161.25	2 080.12

PSA - Probabilistic sensitivity analysis
All currency in local currency unit - Bangladeshi Taka (BDT). Costs are inflation unadjusted values.
PSA values can be changed on each simulation based on the respective distribution drawn from mean and variance.

Table S5.4. Input parameters values for probabilistic sensitivity analysis for 2018

Non-cost parameters	PSA Value	Distribution Function	Alpha	Beta	Mean	Variance
Percent sought care from any providers	0.82	Beta	4.98	1.49	0.77	0.02
Percent not sought care from any providers	0.18	Beta	19.02	63.68	0.23	0.00
Percent sought care from informal sector	0.13	Beta	22.32	194.40	0.10	0.00
Percent sought care from pharmacies	0.50	Beta	11.84	11.56	0.51	0.01
Percent received homecare	0.22	Beta	18.34	53.31	0.26	0.00
Percent sought care from health facility	0.37	Beta	13.59	17.36	0.44	0.01
Percent of outpatient cases	0.96	Beta	3.04	0.13	0.96	0.01
Percent of hospitalized cases facility	0.04	Beta	23.96	575.04	0.04	0.00
Labor force participation rate	0.61	Beta	503.60	316.59	0.61	0.00
Unemployment rate	0.05	Beta	6.61	147.22	0.04	0.00
**Cost parameters (Cost per diarrhoea episode)**	**PSA Value**	**Distribution Function**	**Alpha**	**Beta**	**Mean**	**Variance**
Informal care case	1 055.51	Gamma	12.80	88.58	1 133.89	100 442.24
Pharmacy case	1 292.76	Gamma	12.50	99.50	1 243.80	123 762.41
Homecare case	1 086.61	Gamma	6.24	131.68	821.74	108 208.25
Outpatient case	1 752.70	Gamma	10.33	168.09	1 736.34	291 853.09
Inpatient case	5 945.72	Gamma	17.60	254.68	4 483.65	1 141 911.11
No care case	742.98	Gamma	12.50	57.33	716.67	41 088.89

PSA - Probabilistic sensitivity analysis
All currency in local currency unit - Bangladeshi Taka (BDT).
PSA values can be changed on each simulation based on respective distribution drawn from mean and variance.

Appendix S6: Cost breakdown

Table S6.1. Annual costs to households and health service providers from diarrhoea in children under-five in Bangladesh in 2007 and 2018 by six care prototypes and percent change in annual costs from 2007 to 2018

Care prototypes	2007 annual costs (million)			2007 annual costs* inflated to 2018 value (million)			2018 annual costs (million)			Percent change from 2007 to 2018		
	Base	Min	Max	Base	Min	Max	Base	Min	Max	Base	Min	Max
Informal care	1 429	1 143	1 714	3 017	2 414	3 621	1 276	1 023	968	-58%	-58%	-73%
Pharmacy	6 551	5 241	7 861	13 838	11 070	16 606	6 877	5 501	8 252	-50%	-50%	-50%
Homecare	1 901	1 521	2 281	4 015	3 212	4 818	2 298	1 648	2 949	-43%	-49%	-39%
Outpatient	5 179	4 208	6 149	10 939	8 888	12 989	7 996	6 237	9 755	-27%	-30%	-25%
Inpatient	504	403	604	1 063	851	1 276	860	715	1 005	-19%	-16%	-21%
No care	502	402	602	1 060	848	1 272	2 339	1 871	2 807	121%	121%	121%
All cases	16 064	12 916	19 212	33 935	27 285	40 585	21 648	16 997	25 738	-36%	-38%	-37%

Min – Minimum estimates, Max – maximum estimates.
All currency in local currency unit – Bangladeshi Taka (BDT) in million.
*2007 costs in 2018 value, inflation adjusted by using general consumer price index 170.16 in 2018 and 80.55 in 2007 [17].

Table S6.2. Cost breakdown of total economic burden of the diarrhoea in children under-five in Bangladesh in 2007 and 2018

Cost component	2007 cost (million)	2007 cost inflated to 2018 value* (million) (%)	2018 cost (million) (%)
Direct cost	6 100	12 886 (13%)	4 828 (8%)
Indirect cost	9 965	21 049 (21%)	16 820 (26%)
Economic loss from premature deaths	31 726	67 018 (66%)	42 560 (66%)
Total economic burden	47 791	100 954	64 208

All currency in local currency unit - Bangladeshi Taka (BDT) in million.
*Inflation adjusted by using the general consumer price index 170.16 in 2018 and 80.55 in 2007 [17].

References

1 Zimmermann M, Kotloff K, Nasrin D, Roose A, Levine MM, Rheingans R, et al. Household costs of diarrhea by etiology in 7 countries, The Global Enterics Mulitcenter Study (GEMS). Open Forum Infect Dis. 2019;6(4):ofz150.

2 Hasan MZ, Mehdi GG, De Broucker G, Ahmed S, Ali MW, Martin Del Campo J, et al. The economic burden of diarrhea in children under 5 years in Bangladesh. International Journal of Infectious Diseases. 2021;107:37–46.

3 Lamberti LM, Fischer Walker CL, Black RE. Systematic review of diarrhea duration and severity in children and adults in low- and middle-income countries. BMC Public Health. 2012 Apr 6;12(1):1–11.

4 International Labour Office (ILO). Decent work country profile Bangladesh. 2013. Available: www.ilo.org/publns. Accessed: 26 February 2022.

5 Ministry of Labour and Employment Bangladesh. Bangladesh Minimum Wages. 2021. Available: https://tradingeconomics.com/bangladesh/minimum-wages. Accessed: 15 January 2022.

6 Gates Enterics Project & University of Maryland. Study GEMS1 HUAS/HUAS Lite Survey. ClinEpiDB rel. 7. 2019. Available: https://clinepidb.org/ce/app/workspace/analyses/DS_221d2bcac4/new/details. Accessed: 10 March 2022.

7 Alam MJ. Prevalence and costs of childhood diarrhoea in the slums of Dhaka. 2009. Available: https://opendocs.ids.ac.uk/opendocs/handle/20.500.12413/4430. Accessed: 25 January 2022.

8 World Health Organization (WHO). Econometric estimation of WHO-CHOICE country-specific costs for inpatient and outpatient health service delivery. 2021. Available: https://www.who.int/publications/m/item/who-choice-estimates-of-cost-for-inpatient-and-outpatient-health-service-delivery. Accessed: 18 December 2021.

9 World Bank's Water and Sanitation Program. Flagship report: Economic impacts of inadequate sanitation in Bangladesh. 2012. Available: https://documents1.worldbank.org/curated/en/379261468210888740/pdf/717330WP0Box370SI0Bangladesh0Report.pdf. Accessed: 30 January 2022.

10 Pavel MS, Chakrabarty S, Gow J. Cost of illness for outpatients attending public and private hospitals in Bangladesh. Int J Equity Health. 2016;15(1):167.

11 Ali, Q. L. Calculation of total unit cost for diarrhoeal management at district hospital and Thana health complex. 2001. Available: http://oldweb.heu.gov.bd/pdf/Research Paper No.20.pdf. Accessed: 13 March 2022.

12 Bhuiyan MU, Luby SP, Alamgir NI, Homaira N, Mamun AA, Khan JAM, et al. Economic burden of influenza-associated hospitalizations and outpatient visits in Bangladesh during 2010. Influenza Other Respir Viruses. 2014;8(4):406–13.

13 Rheingans R, Kukla M, Faruque ASG, Sur D, Zaidi AKM, Nasrin D, et al. Determinants of household costs associated with childhood diarrhea in 3 South Asian settings. Clin Infect Dis. 2012;55(Suppl 4):S327-35.

14 Tahsina T, Ali NB, Hoque DME, Huda TM, Salam SS, Hasan MM, et al. Out-of-pocket expenditure for seeking health care for sick children younger than 5 years of age in Bangladesh: Findings from cross-sectional surveys, 2009 and 2012. J Health Popul Nutr. 2017;36(1):1–9.

15 Ahmed S, de Broucker G, Hasan MdZ, Mehdi GG, Martin del Campo J, Constenla D, et al. Cost of diarrhea in children under 5 in Bangladesh (2017-18). Harvard Dataverse, V1, UNF:6:INUNQAM/iu8oOW2iwE/Lnw== [fileUNF]. 13 Feb 2020. Available: https://dataverse.harvard.edu/dataset.xhtml?persistentId=doi:10.7910/DVN/YKPSJ7. Accessed: 15 December 2021.

16 Sultana R, Luby SP, Gurley ES, Rimi NA, Swarna ST, Khan JAM, et al. Cost of illness for severe and non-severe diarrhea borne by households in a low-income urban community of Bangladesh: A cross-sectional study. PLoS Negl Trop Dis. 2021;15(6):e0009439.

17 International Monetary Fund (IMF). IMF Data Access to Macroeconomic & Financial Data - Bangladesh. 2021. Available from: https://data.imf.org/?sk=85b51b5a-b74f-473a-be16-49f1786949b3. Accessed: 26 February 2022.

18 Halder AK, Luby SP, Akhter S, Ghosh PK, Johnston RB, Unicomb L. Incidences and costs of Illness for diarrhea and acute respiratory infections for children < 5 years of age in rural Bangladesh. Am J Trop Med Hyg. 2017;96(4):953–60.

19 Bangladesh Bureau of Statistics, Statistics & Informatics Division, Ministry of Planning. Statistical Yearbook Bangladesh 2020. Dhaka: Bangladesh Bureau of Statistics, Statistics & Informatics Division, Ministry of Planning; 2021.

20 Bangladesh Bureau of Statistics, Statistics & Informatics Division, Ministry of Planning. Statistical Yearbook Bangladesh 2011. Dhaka: Bangladesh Bureau of Statistics, Statistics & Informatics Division, Ministry of Planning; 2012.

21 United Nations, Department of Economic and Social Affairs, Population Division. World Population Prospects 2019, Online Edition. Rev.1., archive. 2019. Available: https://population.un.org/wpp/Download/Archive/Standard/. Accessed: 15 December

2021.

22 National Institute of Population Research and Training (NIPORT), Mitra and Associates & Macro International. Bangladesh Demographic and Health Survey 2007. Dhaka and Calverton: NIPORT, Mitra and Associates, and Macro International; 2009.

23 National Institute of Population Research and Training (NIPORT) & International Coaching Federation (ICF International). Bangladesh Demographic and Health Survey 2017-18. Dhaka, Bangladesh, and Rockville, Maryland, USA: NIPORT and ICF; 2020.

24 Nasrin D, Wu Y, Blackwelder WC, Farag TH, Saha D, Sow SO, et al. Health care seeking for childhood diarrhea in developing countries: Evidence from seven sites in Africa and Asia. Am J Trop Med Hyg. 2013;89:3–12.

25 Rafferty ER, Schurer JM, Arndt MB, Choy RKMM, De Hostos EL, Shoultz D, et al. Pediatric cryptosporidiosis: An evaluation of health care and societal costs in Peru, Bangladesh and Kenya. PLoS One. 2017;12(8):e0182820.

26 WHO. The global health observatory: Number of deaths in children aged <5 years, by cause. 2000 [modifed 27 Nov 2018]. Available: https://www.who.int/data/gho/data/indicators/indicator-details/GHO/number-of-deaths Accessed: 15 December 2021.

27 Government of the People's Republic of Bangladesh, Trading Economics. Bangladesh Retirement Age - 2011-2020 Historical. 2020. Available: https://tradingeconomics.com/bangladesh/retirement-age-men. Accessed: 15 December 2021.

28 International Labour Organization (ILO). Labor force participation rate, total (% of total population ages 15-64) (modeled ILO estimate) - Bangladesh | Data. 2021. Available: https://data.worldbank.org/indicator/SL.TLF.ACTI.ZS?%202020.&%20maxi%20value%20between%201991&locations=BD. Accessed: 30 December 2021.

29 International Labour Organization (ILO). Unemployment, total (% of total labor force) (modeled ILO estimate) - Bangladesh | Data. World Bank. 2021.https://data.worldbank.org/indicator/SL.UEM.TOTL.ZS?%202020.&%20maxi%20value%20between%201991&locations=BD. Accessed: 30 December 2021.

30 Hossai MZ, Islam MS, Rahaman MdM. Capturing market share and creating brand image: The scenario of mobile phone sets industry in Bangladesh. Journal of Business Studies Quarterly. 2017;8(4):2152-1034.

31 Bangladesh Bureau of Statistics, Trading Economics. Bangladesh average monthly income. 2021. Available: https://tradingeconomics.com/bangladesh/wages. Accessed: 15 January 2022.

32 Bangladesh Bureau of Statistics. Consumer price index, inflation rate and Wage rate index, Bangladesh. Dhaka: Bangladesh Bureau of Statistics; 2019.

33 Haacker M, Hallett TB, Atun R. On discount rates for economic evaluations in global health. Health Policy Plan. 2020;35(1):107–14.

34 Black R, Fontaine O, Lamberti L, Bhan M, Huicho L, El Arifeen S, et al. Drivers of the reduction in childhood diarrhea mortality 1980-2015 and interventions to eliminate preventable diarrhea deaths by 2030. J Glob Health. 2019;9(2): 020801.
